# Building new twenty-first century medical school libraries from the ground up: challenges, experiences, and lessons learned

**DOI:** 10.5195/jmla.2019.493

**Published:** 2019-01-01

**Authors:** Nadine Dexter, Joanne M. Muellenbach, Elizabeth R. Lorbeer, Debra Rand, Matthew E. Wilcox, Bradley A. Long

**Affiliations:** Director, Harriet F. Ginsburg Health Sciences Library, University of Central Florida College of Medicine, Orlando, FL, nadine.dexter@ucf.edu; Director, Jay Sexter Library, Touro University Nevada, Henderson, NV, joanne.muellenbach@tun.touro.edu; Director, Library, Western Michigan University Homer Stryker M.D. School of Medicine, Kalamazoo, MI, elizabeth.lorbeer@med.wmich.edu; Associate Dean for Library Services, Donald and Barbara Zucker School of Medicine at Hofstra/Northwell, Hempstead, NY, debra.rand@hofstra.edu; Director, Edward and Barbara Netter Library, Quinnipiac University, Hamden, CT, matthew.wilcox@qu.edu; Embedded Health Sciences Librarian, Harrell Health Sciences Library, University Park Program, Penn State University, State College, PA, bal56@psu.edu

## Abstract

The twenty-first century library at a newly opened medical school often differs from those at traditional medical schools. One obvious difference is that the new medical school library tends to be a born-digital library, meaning that the library collection is almost exclusively digital. However, the unique issues related to building a library at a new medical school are not limited to online collections. A unique start-up culture is prevalent, of which newly appointed directors and other library and medical school leaders need to be aware. This special paper provides an overview of best practices experienced in building new medical school libraries from the ground up. The focus is on the key areas faced in a start-up environment, such as budgeting for online collections, space planning, staffing, medical informatics instruction, and library-specific accreditation issues for both allopathic and osteopathic institutions.

## INTRODUCTION

Since the turn of the century, there has been a significant increase in the number of allopathic and osteopathic medical schools that have received their initial accreditation as well as medical libraries to support them. An obvious trend in the libraries of these new medical schools is their focus on digital collections, rather than on collections in traditional print format. In fact, these new medical libraries tend to be born-digital, with collections that are almost exclusively online. The start-up culture for such born-digital medical libraries is unique, as each library must consider the special needs of its institution, its medical school curriculum, and its community, while operating within the budget and space provided. While each medical library has been tailored to meet the needs of its respective new medical school, several themes have emerged that are common in new born-digital library implementations.

This paper evolved in response to the increasing number of library leaders in new medical schools seeking advice on how to build born-digital libraries from the ground up. In addition, experiences shared amongst the new medical school library leaders can also be relevant for library directors who are new to their roles, librarians who are at new regional campuses, and already established library directors who manage more traditional brick-and-mortar medical school libraries. The authors are library directors, library deans, and other library leaders who have leadership experience in one or more start-up medical libraries in medical schools of various types, including allopathic and osteopathic, independent and preexisting, and private and public university settings.

## BACKGROUND

The Association of American Medical Colleges (AAMC) predicts that as the population increases and ages, the current supply of physicians will not meet the demand [1]. Although the number of physicians is on the rise, it will not be sufficient to alleviate the shortage of tens of thousands of doctors that is anticipated over the next decade. Since 2000, there has been a significant increase in the number of new medical schools and, with them, new medical libraries [2]. Library leadership for these twenty-first century medical schools has had to consider the needs of a new generation of library users who have grown up in the digital age when making decisions related to key areas such as space planning, staffing, budgets and collections, accreditation through the Liaison Committee on Medical Education (LCME) or the Commission on Osteopathic College Accreditation (COCA), and medical informatics instruction.

Born-digital is a relatively new concept when applied to medical school libraries [3]. The first born-digital health sciences library that was designed to be 100% electronic was created for Florida State University (FSU) College of Medicine in 2000 [4]. Between 1982 and 2000, no new allopathic medical schools were accredited, and only 4 new osteopathic schools were accredited in the United States and Canada. Since 2000, 48 new medical schools, both allopathic and osteopathic, have received initial accreditation [5, 6], and many of their library directors have followed the lead of the FSU Medical Library. These new medical libraries have tailored the resources and services that they provide based on such factors as the local health care needs, the type of medical school they support, and the design of their medical school curricula.

A growing trend of medical school library users has been to access library collections—including books, journals, databases, and other clinical tools—via mobile devices. Therefore, these new libraries need to be available “anywhere, anytime, on any device,” the motto of the University of Central Florida College of Medicine (UCFCOM) Harriet F. Ginsburg Health Sciences Library. This means that web pages need to be agile and available on a variety of screen sizes. In addition, since the collections and space of many born-digital medical libraries may not be visible or otherwise resemble a traditional library, marketing of library resources and services becomes increasingly important. The born-digital librarian needs to describe through effective promotional methods their website offerings, and, thus, the development of a marketing plan is imperative. Furthermore, the cultivation of digital library champions can be developed through effective use of social media and scheduling of events [7].

During the first decade of the twenty-first century, a small group of library directors for new medical schools who were all members of the Association of Academic Health Sciences Libraries (AAHSL) came together to share ideas, give advice, and provide support related to their born-digital libraries. AAHSL comprises the libraries serving the accredited US and Canadian medical schools affiliated with the AAMC. Over time, these library directors for new medical schools created an informal discussion list and a blog, and collaborated on conference posters.

In 2011, the library directors developed an initial needs assessment survey to identify the major challenges faced by library directors for new medical schools. The following year, in response to requests from these new library directors for more informed assistance, a two-day AAHSL symposium was planned and hosted at the UCFCOM. The response to and attendance at the symposium was so positive that, in 2013, it led to the formation of the AAHSL New and Developing Academic Health Sciences Libraries (NDAHSL) Committee. One of the committee’s first initiatives was to review the results of the 2011 needs assessment survey and, in 2015, to conduct a follow-up survey to determine whether the needs of the directors and leaders of these new medical school libraries had changed since the initial survey. Survey results confirmed that topics of vital interest to library leaders in new medical school libraries included space planning, staffing, budgets and collections, LCME and COCA accreditation, and medical informatics instruction. In addressing these topics, we will focus on best practices, as well as our lived experiences as trailblazers in born-digital libraries.

## SPACE PLANNING

In general, born-digital academic health sciences libraries tend to have a smaller footprint, as they are almost 100% print-free. They largely consist of flexible “open” space, with a variety of work surfaces and study spaces, and plenty of electrical outlets and WiFi connections to meet user needs for concentration, collaboration, contemplation, communication, and socialization [8]. Therefore, space planning for born-digital libraries should begin with a projection of the user population and their needs over time. Library planners should consider how the library functions relative to other campus libraries and affiliated and owned hospitals. They should also consider the types and quantity of individual or group study rooms throughout the building where the library is located, the mix of funding sources, the political climate on campus, the available space, and programmatic needs [9].

In addition, library planning should be aligned with institutional priorities [10]. For instance, does the school leadership want the library study space to be accessible to other campus students who are not the library’s primary users? Finally, planners must be knowledgeable about trends in library architecture and be flexible. The following specific factors should be considered when space planning for born-digital libraries:

1. A decision about the need for print collections is an important first step. Space should be flexible, so that stacks can be removed and the space repurposed as needed. In some cases, there might not be a need for any book stacks but rather just a small, secure space for a reserve collection.
2. An information commons with public access computers, laptops, and tablets available for loan; printers; and charging stations is recommended. A combination of WiFi and hardwired infrastructure offers the most flexibility to accommodate future changes.
3. A multimedia lab should be considered, with partners throughout the school identified to help comanage the space. New technology may be needed for e-course materials, graphics, research data, scanning, streaming video, video conferencing, and virtual reality learning tools. Additionally, some schools are experimenting with makerspaces and 3D print and scanning technology. As newer technologies emerge and are adopted, the lab space and its furnishings need to be flexible to easily accommodate newer equipment.
4. Small group study space is needed to accommodate active learning styles and interdisciplinary collaboration, but individual study spaces remain essential. A combination of tables, carrels, individual study rooms, and comfortable lounge seating, with whiteboards and network connections, is recommended. Most people desire natural light for quiet reflection and study, so arranging study space along the outer edges of the building, with service areas arranged in the center should be considered.
5. Students need 24/7 access to both health sciences library spaces and online content. Staff offices and space for reserve or historical collections should be designed so that they can be closed off outside of staffed hours.
6. Librarians are providing more educational programs in information literacy, evidence-based medicine, and bioinformatics—often within the formal curricula. While a dedicated computer training lab is nice to have, there is a greater focus on access to teaching space with sufficient WiFi capability for users to bring their multiple devices.
7. Faculty and health professionals often prefer to access library resources remotely through their mobile devices, while students tend to be more frequent users of library space. In general, students desire social areas, so it would be beneficial to plan for a café in or near the library, if one is not available elsewhere in the building. Traditionally, many libraries have not allowed food or drink in their space. A more flexible policy with an understanding of student responsibility may encourage students to use the library as their primary study space and facilitate a positive attitude toward library services.
8. As the demand for consumer health information increases, community members might represent a distinct proportion of library users. Planning should involve decisions on the number of public access computers, their location, and the range of consumer resources that are provided.
9. Decisions regarding historical collections may arise, as these materials have special appeal for faculty, alumni, and health professionals who can be advocates for the library. A well-planned history of medicine, archives, or rare books area should include an exhibits space that can be used for special events that appeal to library donors or supporters.

Overall, born-digital library space planning should strive for excellence in the availability of high-tech resources and flexible study spaces that serve the specific needs of the user population. It should promote the library as a showplace for the campus and as a center for scholarly activity. Finally, born-digital library space planning should reflect a community’s vision of itself, where information is available at the user’s fingertips.

## STAFFING

Staffing and structure vary in born-digital libraries, but a common trait is a smaller number of library staff. The 40th edition of the *AAHSL Annual Statistics of Medical School Libraries in the United States and Canada* reported that the average number of librarian/professional positions in member libraries was 9.74 full-time equivalents (FTEs); for 21st century born-digital libraries that reported their data, this number was 4.00 FTEs [11]. This small number of staff must navigate the complex education, research, and patient care needs of new and emerging schools and clinics. Each of these new medical schools starts with a clean slate, bound only by LCME or COCA requirements, and works to be innovative in how it delivers medical education and manages the student clerkship experience. Library staff members must be comfortable in this new environment and quick to react to the considerable access they have to the medical school curriculum and faculty.

Organizational charts vary with the type of library but are typically flat, with the existence of several different staffing models. Some new medical libraries have their own dedicated space and staff. Other new medical schools, instead of developing an entirely new library, expand on existing university libraries to provide medical subject specialists to assist medical library users. Subject specialists are usually placed into the role of jack-of-all-trades, providing all levels of public services, single-handedly integrating information literacy into the curriculum, and developing the online medical collection, while also being responsible for the library portion of medical school accreditation. These specialists often serve as embedded departmental liaisons, placed physically within the medical school without a traditional physical library space. They may also serve from within the university library, at a distance from the medical school.

The primary advantage of employing a medical subject specialist is that the university library can provide more cost-effective services by utilizing already existing departments and services, such as acquisitions, course reserves, interlibrary loan, and web services. The medical subject specialist may also benefit by working in close proximity to nursing, allied health, and science, technology, engineering, and math (STEM) librarians [12]. However, a disadvantage is that these librarians can feel isolated and overworked. They might also find themselves in the middle of highly political situations that would be better handled by a dedicated health sciences library director. Existing university library administrators are often very willing to assist but often lack the background and experience in working with medical school administrators. Also, there is concern that the medical school could decrease its financial support if there are other preexisting nursing or allied health programs. In general, it is preferable to have dedicated staff with a director or dean as head of the medical library.

In this new age of born-digital libraries, the traditional librarian position merges with those of information technology professional, administrator, educator, and bioinformatician. Thus, defining roles, identifying good candidates, and defining library patrons can become challenging. In 2005, Donald A. B. Lindberg and Betsy L. Humphreys, FMLA, published a thought-provoking and uncannily accurate article in the *New England Journal of Medicine* predicting the solutions to these problems. While their article was published more than ten years ago, their “evolutionary scenario for the medical libraries of 2015” resonates with crystal ball clarity by describing things like “information specialist, bioinformatics, [and] database specialist” for the new roles they saw for medical librarians [13]. Indeed, in terms of staffing, what is needed for born-digital libraries is a kind of hybrid librarian who can be a contract negotiator, PubMed educator, systematic reviewer, and teacher all rolled into one overworked medical librarian. Librarians who are creative, technologically savvy, knowledgeable about evidence-based medicine, problem-solvers, and expert multitaskers are in high demand at born-digital medical libraries. Major areas of traditional library services—such as acquisitions, cataloging, interlibrary loans, and network/systems coordination—are frequently outsourced to the main library as part of a shared services agreement.

The Medical Library Association’s Academy of Health Information Professionals (AHIP) is important in this new environment. Many new medical libraries will request that librarians have at least their preliminary membership by the third year of hire. Academy membership is helpful for ensuring professional development at institutions that do not have a formal librarian promotion or tenure system. Academy membership is becoming a very useful tool that can help new institutions keep up with current trends and issues. In addition, for staff members without a traditional library degree or certification, having a background in a health sciences–related field can be invaluable.

Small staff sizes and new roles are just a few of the challenges facing born-digital medical libraries. However, this could be an opportunity to develop into new roles and to work collaboratively with administration and hospital partners to create a new academic environment where librarians are part of the team bent on creating a new leaner, more fluid academic medical education model.

## BUDGETING FOR AND MANAGEMENT OF COLLECTIONS

The variety of resources and formats that are available for born-digital libraries can be overwhelming. In addition to e-books, e-journals, article databases, and clinical decision–support point-of-care tools, there are anatomical models, geographic information system (GIS) tools, image databases, United States Medical Licensing Examination (USMLE) board review materials, and video streaming services, all of which need to be accessed on a variety of devices, including desktops, laptops, smartphones, and tablets. Planning for an “opening day” collection in a new medical library that fits within the library’s current budget requirements can present a challenge. Being able to develop the budget over time, after identifying the organization’s collection needs, is a huge advantage. Additionally, it is critical to determine how to manage the budget and library collection over time, to determine how to best discover new resources, and to understand the institution’s budget process. Furthermore, since the library’s budget and collections are a major focus for the LCME and COCA, developing and managing these resources in an organized and documented fashion is essential.

The library budget will determine which resources the library can provide. Some institutions may define specific categories of need in the budget—such as books, journals, databases, document delivery, and institutional memberships—while other budgets may be completely open for the library director to define [14]. When developing the budget, the library director should think long term. For example, with a five-year budget, the director should consider that curriculum content may evolve during the first few years and that there may be a need for a budget cushion to account for additional curricular resources. In addition, a newly created library will be a “new” customer to vendors, which is often associated with a “discounted” introductory price that should be considered during the initial budget request.

While the collections should reflect the institution’s overall mission, there should be a strong curriculum focus. Although collections will be primarily electronic, library directors should plan to budget for some print, as students prefer having print copies of required titles for studying. To help shape the library collection, library directors should:

1. schedule telephone interviews with the newly established medical school libraries that most closely match their user profile for firsthand advice
2. utilize data available from AAHSL to help plan the collections
3. take advantage of collection development tools such as Doody’s Core Titles
4. obtain recommendations from and identify the needs of the course directors, researchers, and other stakeholders
5. be an active member of the education team, participate on curriculum committees, and find faculty champions
6. encourage librarian liaisons to work closely with course directors and key faculty to identify resources and schedule product trials and training for the needed resources

There are several collection management strategies to ensure that new medical school libraries optimize their collections and the impact of their budgets. Cooperative or consortia-based collection agreements can provide the desired resources at the best prices. Consortia can comprise a geographic region, such as the Statewide California Electronic Library Consortium, or cover just one state, such as the Nevada Council of Academic Libraries. They will not only enhance buying power, but library directors will also get extra points from the school’s finance leaders and the LCME or COCA when they report cost-savings. Other strategies to consider include cost-sharing with the parent or affiliate institution, patron-driven acquisitions, and rapid delivery of electronic documents [15].

Once the collections have been selected, the library director will want to select a “discovery” tool or search engine and prominently display it on the main page of the library website to make the collections findable by users. Additionally, the library website should provide links to training and tutorials so that library users can learn how to fully utilize the resources’ features. A full catalog of resources should also be available and obvious to users. The library should plan to track resource usage, to determine the cost-per-use, and to survey users to determine if the resources are meeting user needs and fitting appropriately within the library budget prior to renewing each resource contract.

Because budgeting for an opening day collection in born-digital libraries will emphasize digital resources, which are heavily dependent on technologies, funding will continue to be a critical issue. It is wise to keep up-to-date with trends in scholarly publishing and to support open access initiatives. With appropriate financial support, born-digital libraries and librarians of the future will continue to thrive and serve the expanding needs of medical education.

## ACCREDITATION REQUIREMENTS

### Liaison Committee on Medical Education

The process for a new medical school to achieve full accreditation from the LCME to grant a doctor of medicine (MD) degree is long and complex [16]. Accreditation begins with a medical school applying for LCME candidate status by providing a self-analysis of compliance with the accreditation standards to prove its ability to support a new medical education program. Librarian input on design, sustainability, space, and resources is a critical component of the self-study. Once the medical school progresses from applicant to preliminary status by approval of the LCME, students can be recruited and accepted for the inaugural class. Before the end of the second year of medical school operation, LCME accreditors will visit the campus and conduct their first site visit regarding compliance to grant the medical school with provisional accreditation status. During this process, a set of standards is used to benchmark the medical library staff’s ability to provide resources and services, interact with medical educators and students, and achieve student satisfaction.

The data collection instrument (DCI) is the official documentation tool that is used to prepare for full accreditation surveys. Once the DCI is submitted, there is a three-day site visit by an accreditation team, which is made up of medical educators. This includes a focused review of the DCI and other submitted documents as well as interviews with faculty, students, residents, and the library director.

The DCI includes a standard specific for librarians—“Standard 5.8: Library Resources/Staff”—which is in the “Educational Resources and Infrastructure” category. This standard includes a narrative on student satisfaction with library services, resources, space, hours, collaboration with affiliates, and level of staffing. Standard 5.8B speaks to the ability of the library staff to interact, participate, and deliver content in the medical education program. The inclusion of this standard speaks volumes about the importance and value that the LCME accreditation body places on librarians to be participatory members of curriculum planning committees, governance, and assessment initiatives.

After the site visit, accreditors decide if the school can progress to provisional accreditation status or must correct deficiencies in the program. For schools that progress on to provisional status, another site visit occurs in the fourth year of medical school operation to decide if the program is ready to receive full accreditation status and, therefore, be permitted to graduate its first student class. After a school has received full accreditation, the LCME reviewers can decide to return for reaccreditation site visits as early as two years or up to eight years later [17].

Another DCI standard to which librarians can contribute is “Standard 3.2: Community of Scholars/Research Opportunities,” which asks for a narrative to describe the resources that are available to support medical student participation in research and faculty scholarship. Librarians, who often hold the rank of academic faculty, can provide support to other academic faculty by providing professional development opportunities, including literature search expertise, research methodology, and publication support. Librarians can assist faculty and students with formation of research questions, critical appraisal of biomedical information, and advanced use of research databases and tools. Additionally, librarians can provide support for citation management software, copyright, open access publishing, and scholarly communication.

Librarians can also contribute to “Standard 4.2: Scholarly Productivity.” Librarians may be responsible for providing accurate information on bibliometrics and for tracking faculty publications, even in new schools that are in the process of developing their research programs and that have smaller numbers of publications. The LCME process is now framed within a continuous cycle, so librarians need to submit updated data more often than the once every two- or eight-year accreditation cycle. Annual student evaluations will have a critical role in this process, including their responses to questions about library services.

### Commission on Osteopathic College Accreditation

The American Osteopathic Association (AOA) COCA serves the public by establishing, maintaining, and applying accreditation standards and procedures to ensure that academic quality and continuous quality improvement delivered by colleges and schools of osteopathic medicine (COMs) reflect the evolving practice of osteopathic medicine [18]. The scope of the COCA encompasses the accreditation of COMs. Accreditation action taken by the COCA means a COM has appropriately identified its mission, has secured the resources necessary to accomplish that mission, shows evidence of accomplishing its mission, and demonstrates that it can be expected to continue to accomplish its mission in the future. Accreditation of a COM means that the COM incorporates the science of medicine, the principles and practices of osteopathic manipulative medicine, the art of caring, and the power of touch in a curriculum that recognizes the interrelationship of structure and function for diagnostic and therapeutic purposes, the importance of addressing the body as a whole in disease and health, and the importance of homeostasis and self-regulation in the maintenance of health.

COCA “Standard 3: Facilities, Equipment, and Resources” is specific to librarians [19]. Standard 3.1 states that a COM must have available sufficient and appropriate facilities for the program of instruction to enable students and faculty to successfully pursue the educational goals and curriculum of the COM. Standard 3.2 states that the COM must provide access to appropriate learning resources that are necessary to support the curriculum. The standard 3.2 guideline further states that resources should include, but not be limited to, information technology; student space for individual and group study; and electronic resources, including databases for learning. Standard 3.3 states that the learning resources of all campuses and affiliated teaching sites must be reviewed by the COM to ensure delivery of the curriculum and that COMs should evaluate such sites to ensure that they have the necessary space, technology, and other material as identified by the COM.

For all new medical schools, the road to full accreditation is a journey that takes several years. Library patrons are constantly surveyed for user satisfaction levels. These surveys are one of the benchmarking activities that librarians can develop, as is the use of the library’s statistics and comparison of those statistics with the AAHSL annual statistics. Building and managing a network of library collaborators as the new library grows is also important.

## DEVELOPMENT OF A MEDICAL INFORMATICS PROGRAM

An essential role and responsibility of medical librarians is to provide medical students, faculty, and staff with the skills needed to access, manage, and use library and information resources effectively. In born-digital libraries, librarians have a unique opportunity to prioritize this education from the start by developing a medical informatics program. Librarians play an important role in providing skills to enhance and support lifelong learning that go well beyond medical school. These skills provide a solid foundation for physicians to know how to keep up with the ever-growing body of medical education research literature [20]. Creating a medical informatics curriculum, collaborating with course directors and faculty, and designing and delivering content and assessment techniques are all essential components of born-digital libraries’ medical informatics programs.

In 2014, the AAMC published guidelines that defined activities that all medical students should be able to perform upon entering residency. These core entrustable professional activities (EPAs) offer a practical approach to assessing competence in real-world settings and impact both learners and patients. Medical librarians can play a major role in ensuring that “EPA 7: Form Clinical Questions and Retrieve Evidence to Advance Patient Care” is accomplished. EPA 7 focuses on new residents’ abilities to form clinical questions, retrieve evidence to advance patient care, and apply that evidence to a patient or population [21]. In a medical informatics curriculum, librarians can provide instruction, resources, and activities that emphasize how to formulate answerable clinical questions and how to acquire the appropriate, evidence-based information. Librarians can also help students evaluate the evidence that they find in the literature and ensure that it applies to the clinical problem at hand.

Librarians in new medical schools have a unique opportunity to get in at the ground level and become members of curriculum committees and to develop medical informatics programs. The LCME accreditation body, at all levels of its surveys, asks if the library is involved in curriculum planning and governance. As soon as library directors come onboard, they should develop a deep understanding of the curriculum and get to know the course directors and teaching faculty. Library directors should collaborate with curriculum leaders to establish medical informatics curricula that complement the undergraduate medical education programs’ teaching of evidence-based medicine.

In born-digital libraries, medical informatics is being designed and taught in a variety of methods, including in person, online, asynchronously, or in a blended model. Some programs are integrated into semester-long courses, while others introduce medical informatics during new student orientation with additional components threaded longitudinally over the four years of medical school. Whether the content is focused on demonstrating mobile device apps, point-of-care resources, or PubMed search strategies, it is best to incorporate clinical cases or topics from other courses in the curriculum and to design active, meaningful activities. In this way, students will make the connections, become more engaged, and further their development as lifelong learners. Appropriate timing of medical informatics instruction is critical: it should be delivered at the time of need in the students’ medical education.

Students attach greater significance to medical informatics content that is graded and for which academic credit is awarded. Therefore, library directors should use multiple tools throughout the curriculum for assessing students on their medical informatics knowledge. This will involve locating or creating validated rubrics and using readiness assessment tests, group presentations, role-playing, or journal club projects in addition to standard, multiple-choice, or essay examinations. Library staff should collaborate with course directors to incorporate informatics assessment into post-encounter clinical skills exams, sometimes known as objective structured clinical examinations (OSCEs).

Through the development of a medical informatics program, born-digital libraries have a distinct advantage. They can become involved in curriculum committees early on, while the overall medical curriculum is still under development, and create a medical informatics program that will complement and highlight the unique features of that curriculum. Through collaboration, creativity, flexibility, and persistence, born-digital libraries and librarians at born-digital libraries will continue to thrive and to serve the expanding needs of their medical education and other health sciences programs.

## CONCLUSION

The rise of born-digital medical libraries and the new medical school library directors who manage them is indicative of these challenging times for medical education. In fact, the position of the medical library in the medical school may determine its future growth, support, and management. The “digital natives” or medical students who are the primary users of the born-digital library will continue their journeys with digital content and do it well because they were guided by enthusiastic medical librarians who pioneered what it means to be from a born-digital medical library. Additional challenges for any new institution include identifying the start-up culture and how the library fits within the organizational structure. The reporting structure can have a direct impact on the size of the budget, collections, space, and staff. Additionally, medical school libraries with geographically separate medical campuses need to become proficient in dealing with digital reference services.

These are exciting times for born-digital medical libraries in new, twenty-first century medical schools. The library directors and other library staff who are involved in these endeavors can integrate e-resources and information management knowledge directly into brand-new curricula. Furthermore, the medical librarians can effectively partner with new faculty colleagues and work directly with students to help meet their digital information needs.
